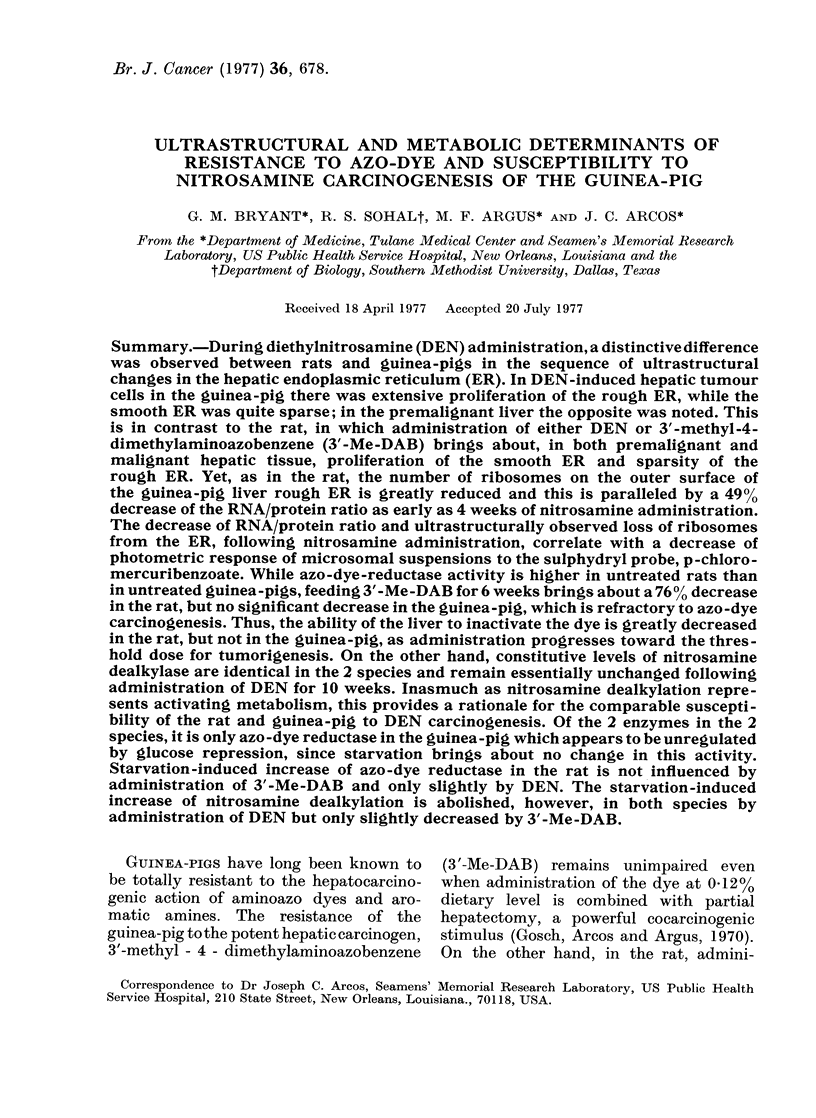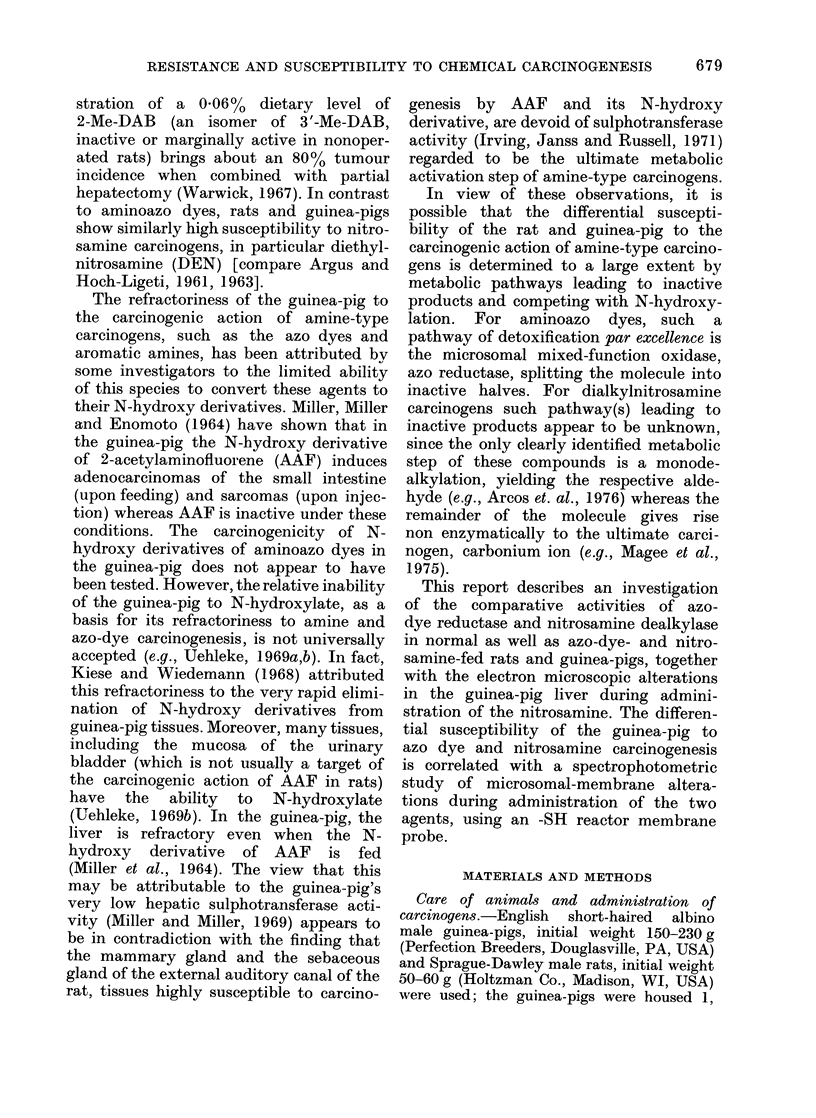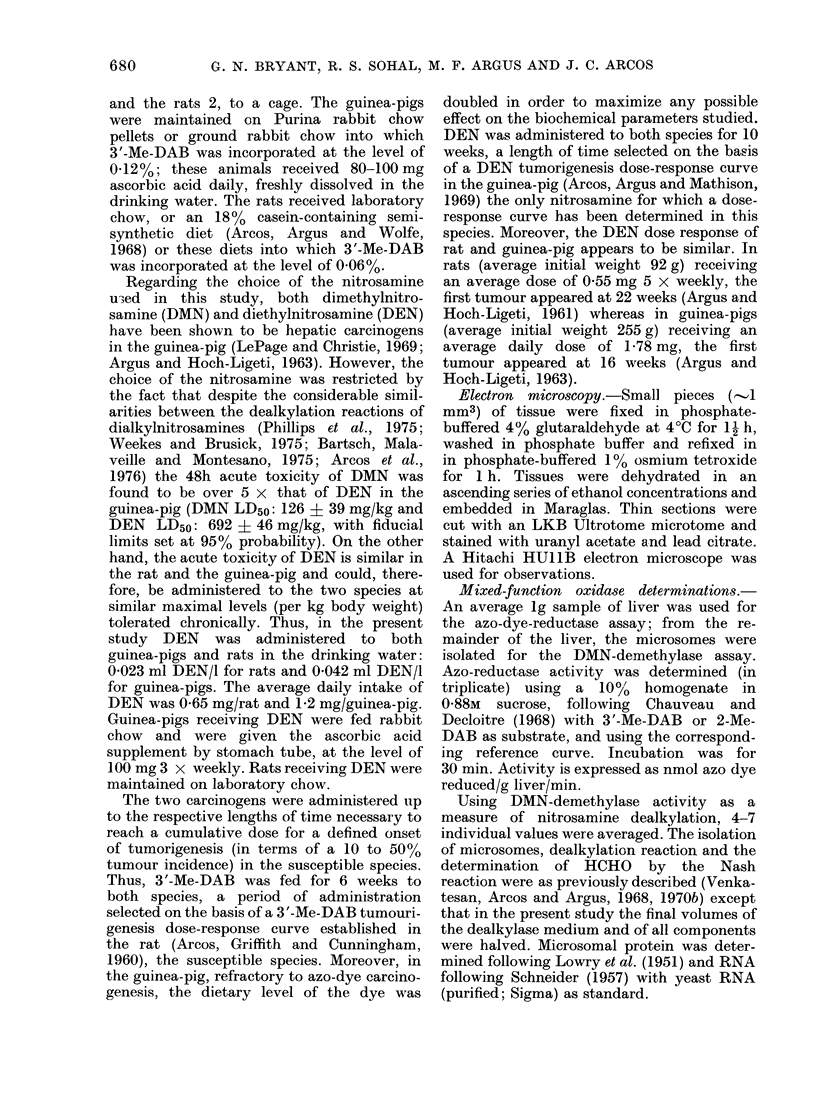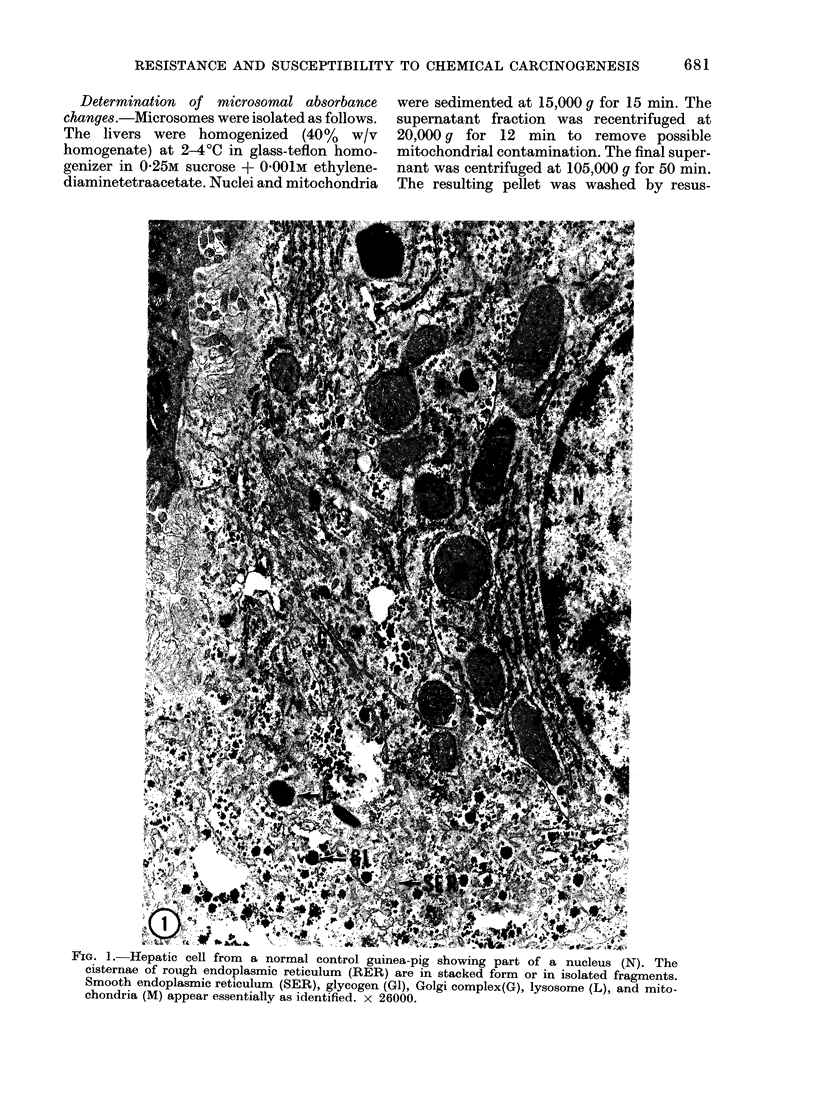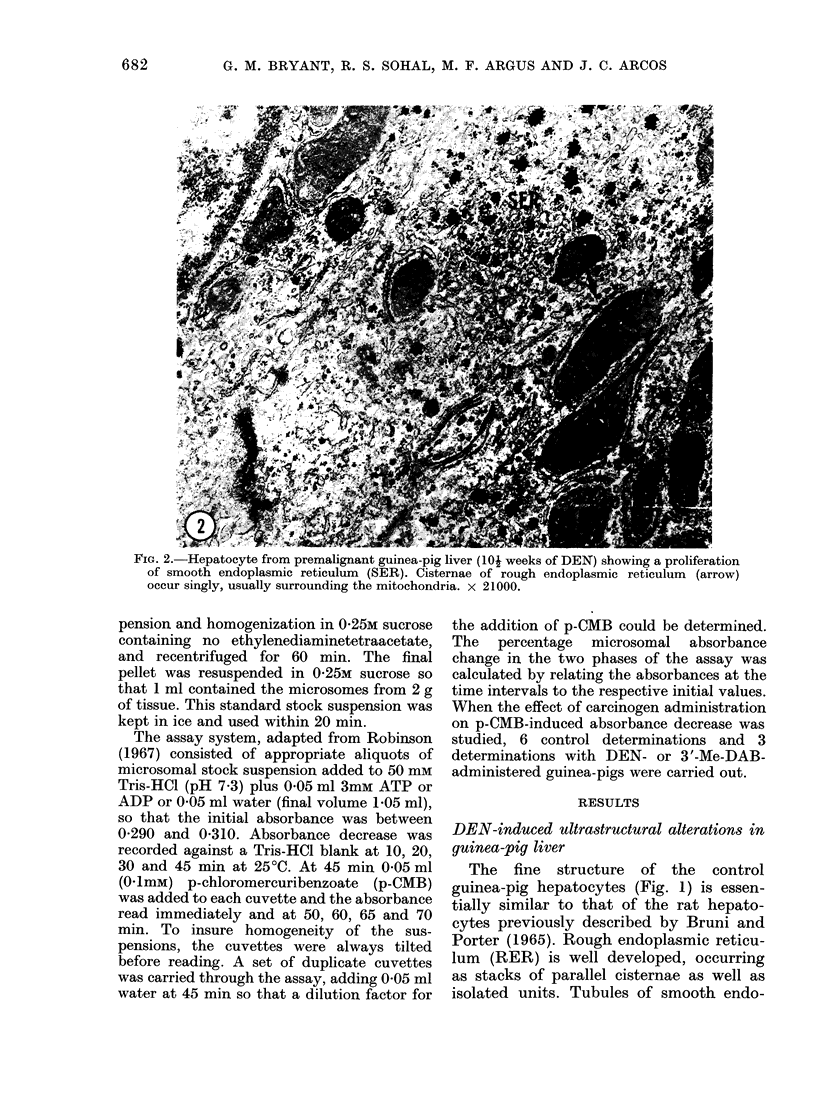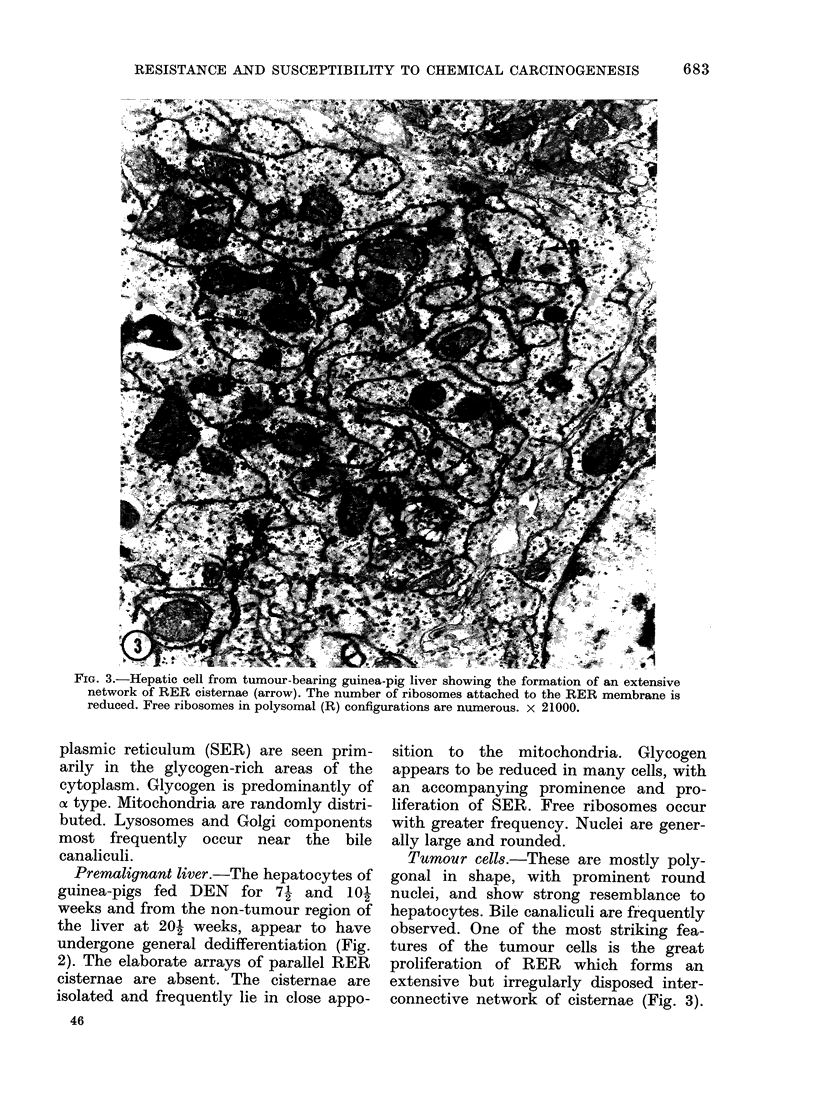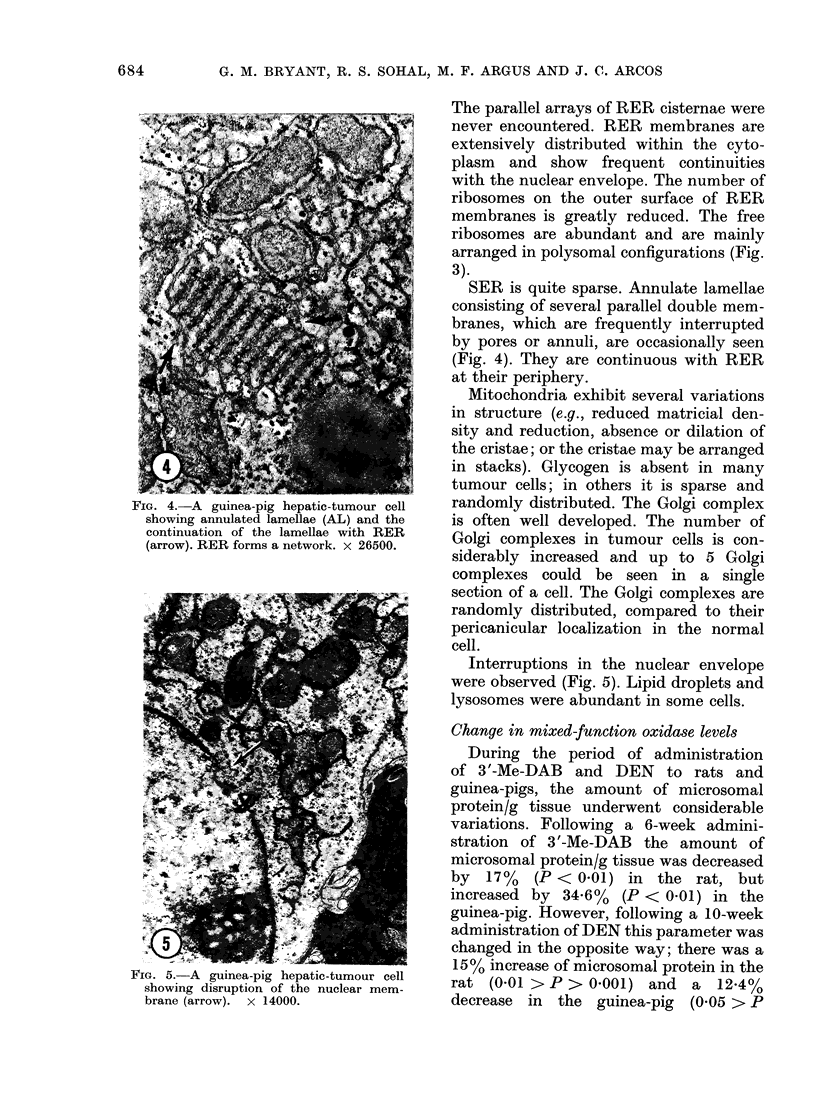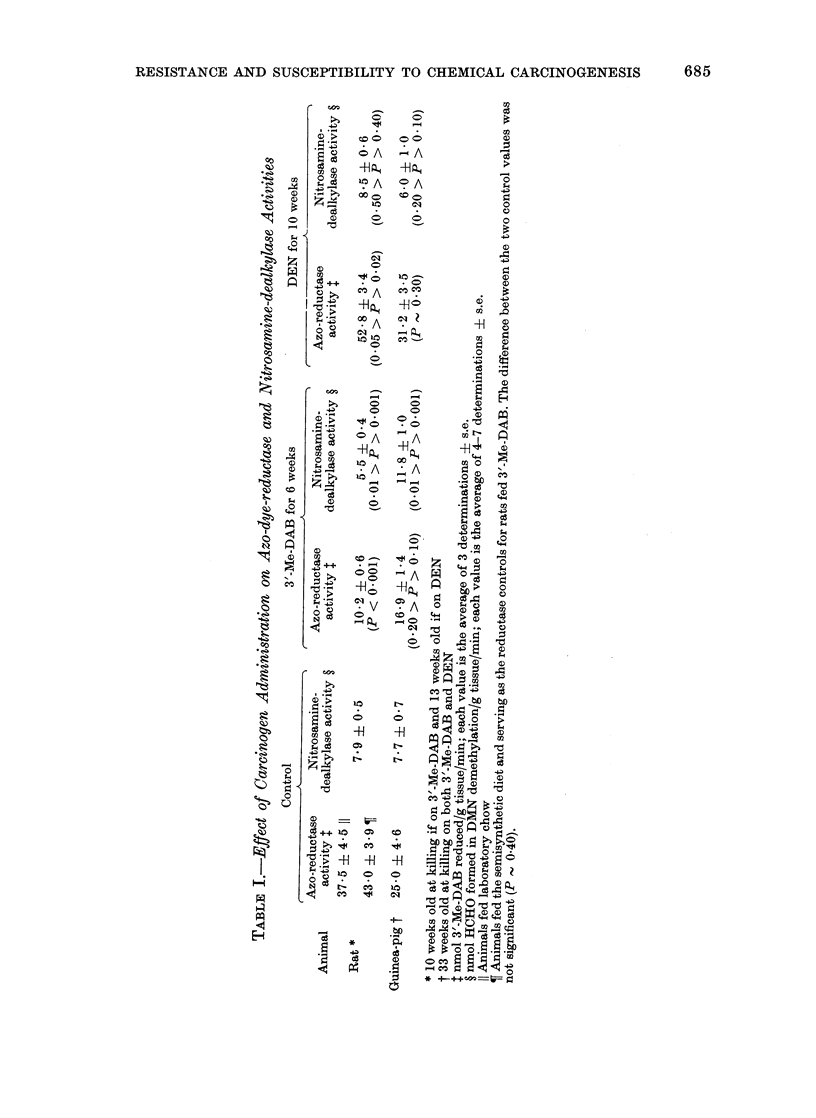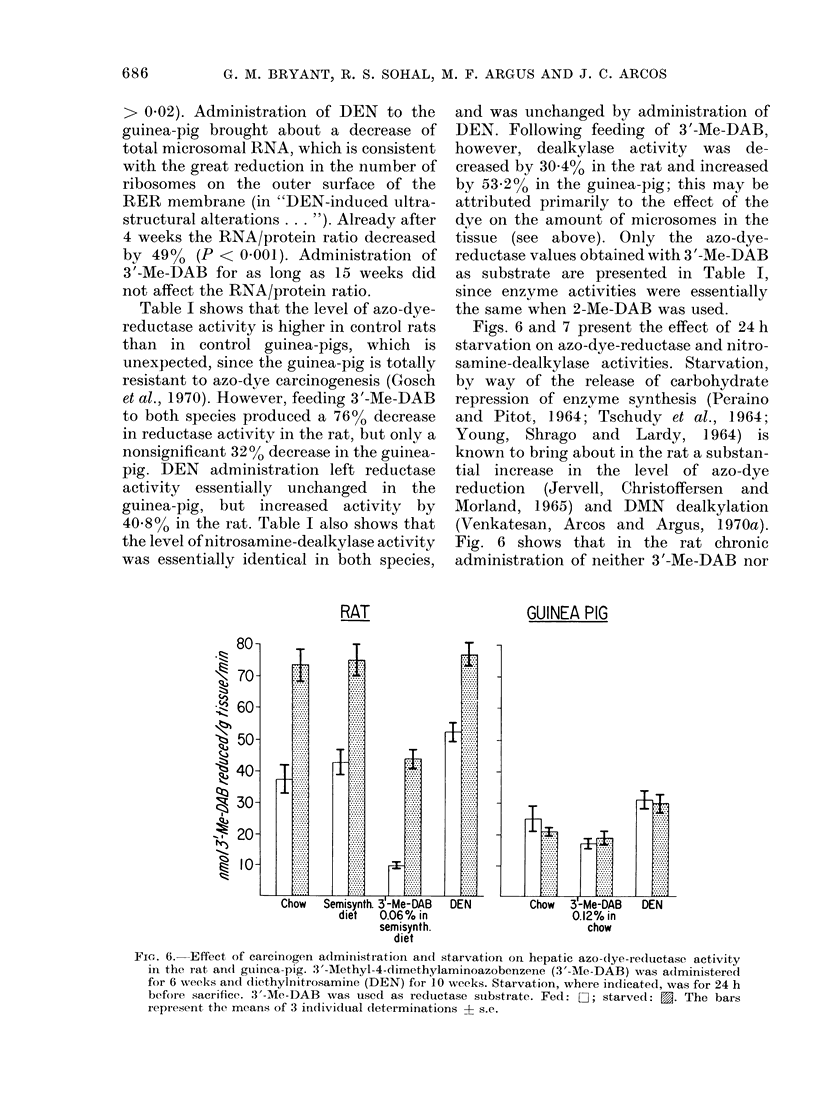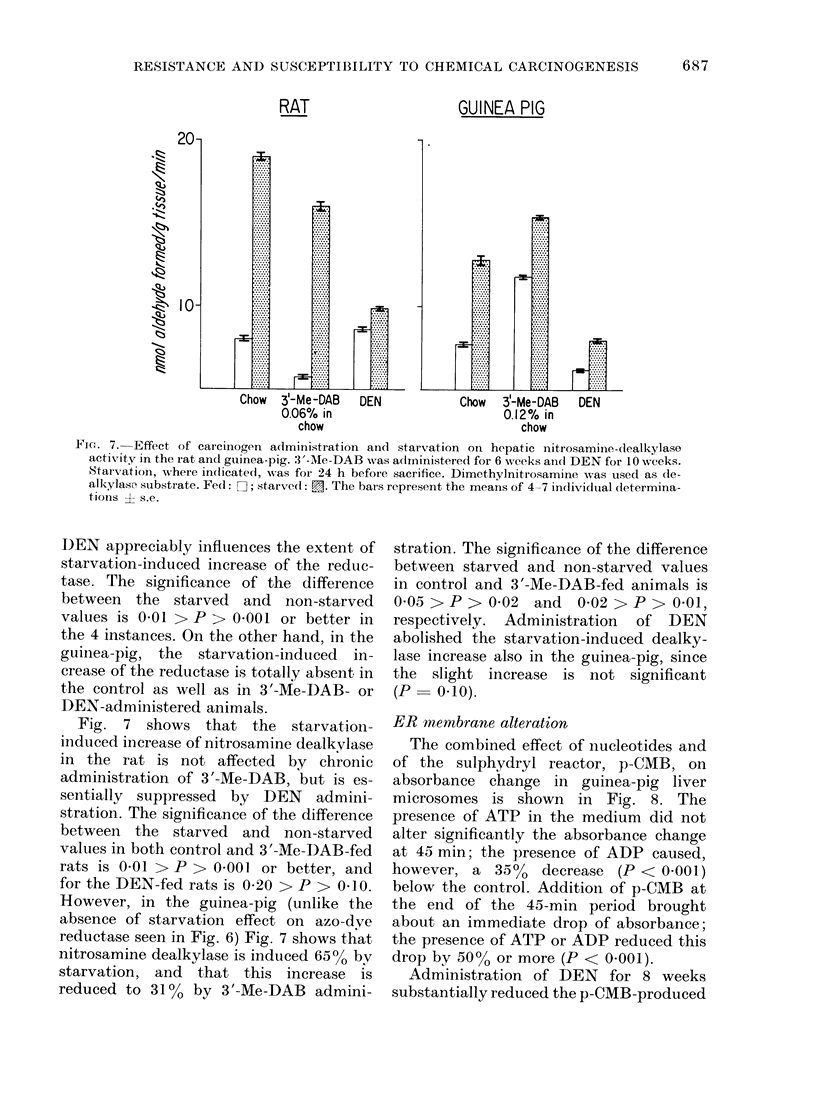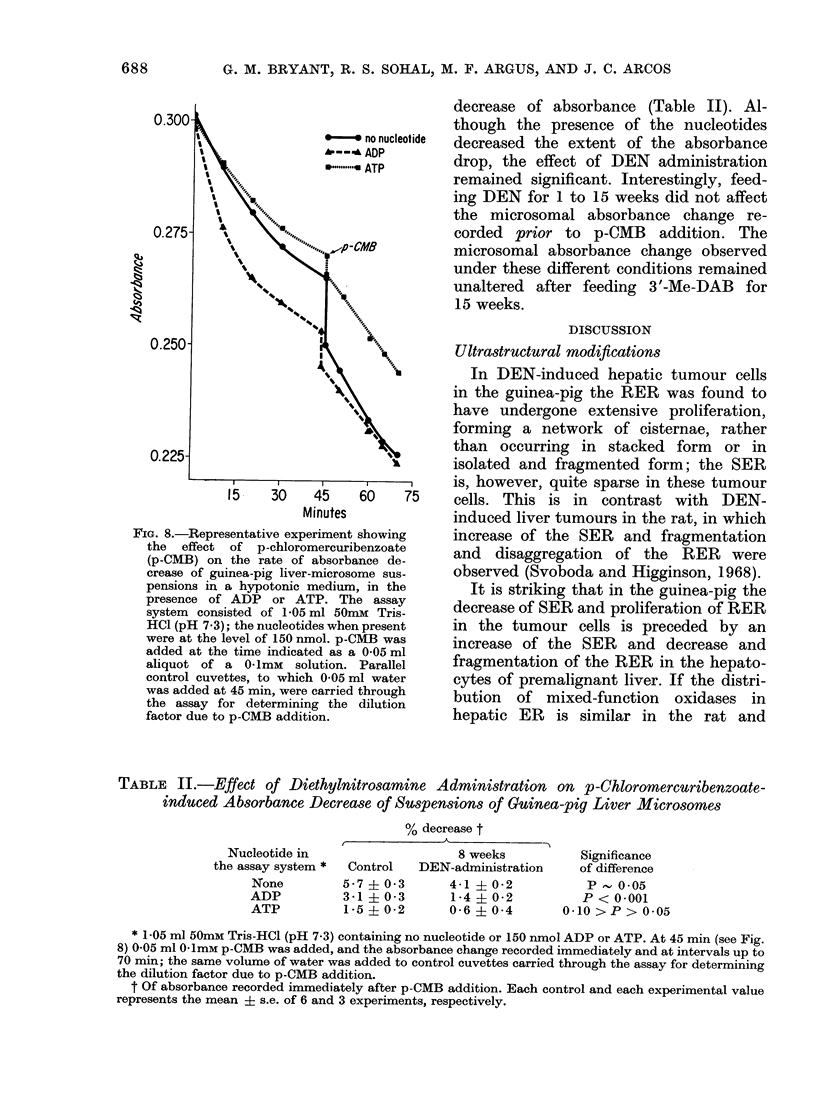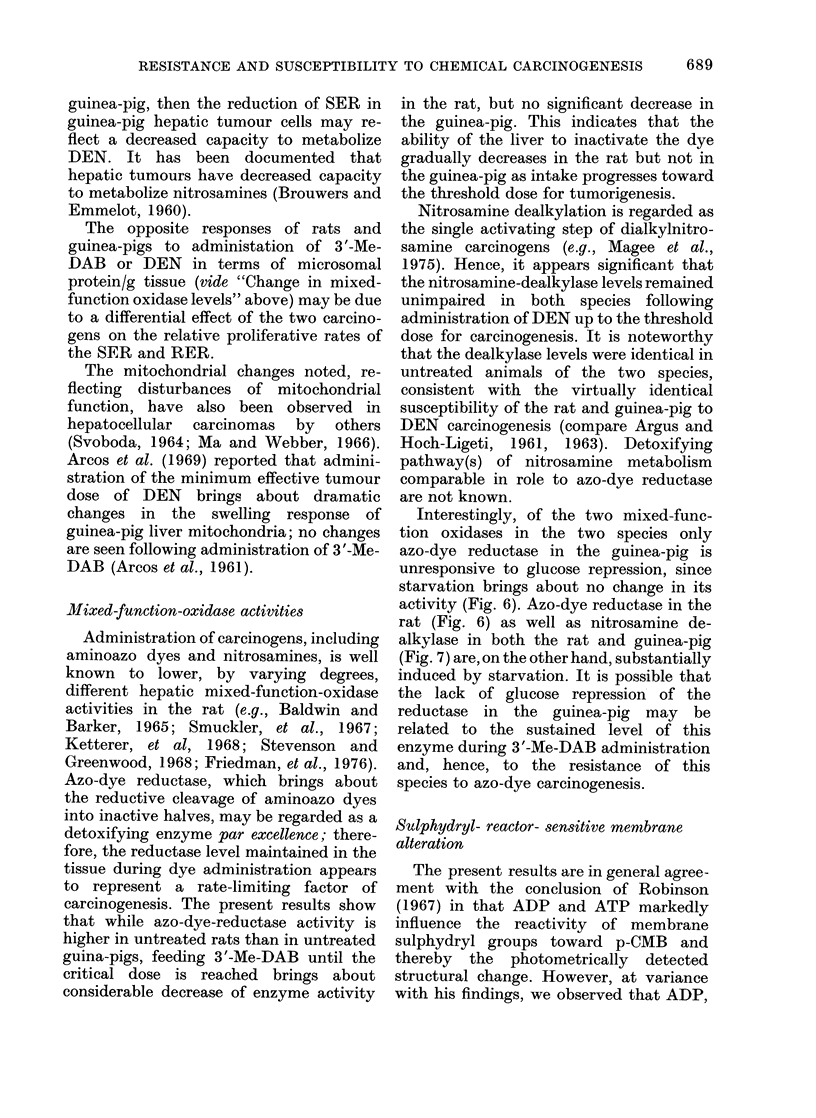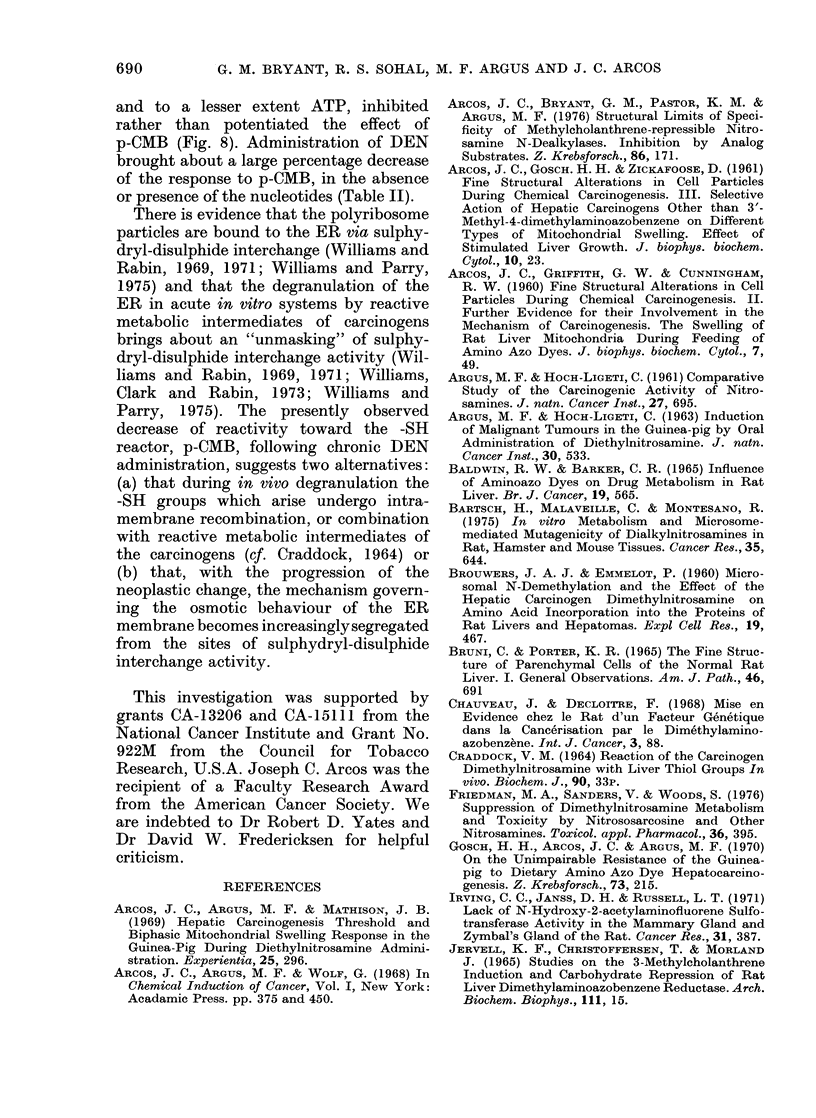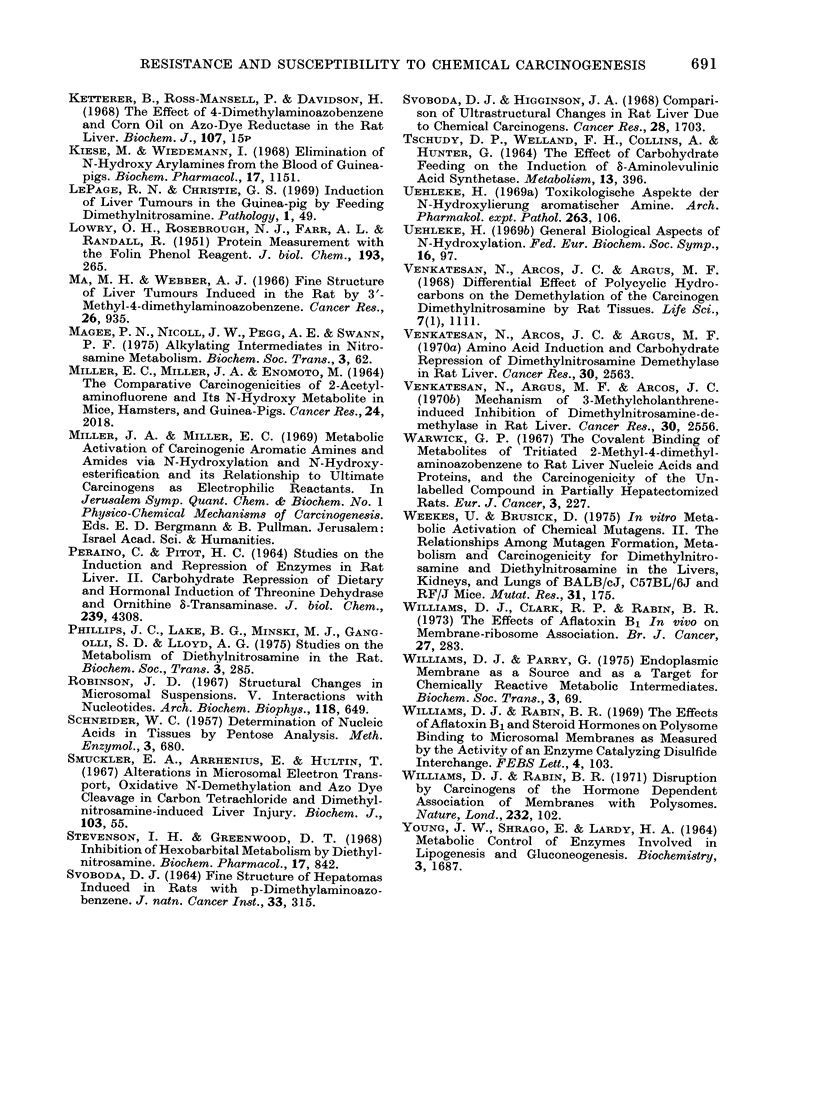# Ultrastructural and Metabolic Determinants of Resistance to Azo-dye and Susceptibility to Nitrosamine Carcinogenesis of the Guinea-pig

**DOI:** 10.1038/bjc.1977.250

**Published:** 1977-12

**Authors:** G. M. Bryant, R. S. Sohal, M. F. Argus, J. C. Arcos

## Abstract

**Images:**


*Br. J. Cancer* (1977) **36**, 678.

### ULTRASTRUCTURAL AND METABOLIC DETERMINANTS OF RESISTANCE TO AZO-DYE AND SUSCEPTIBILITY TO NITROSAMINE CARCINOGENESIS OF THE GUINEA-PIG

G. M. BRYANT*, R. S. SOHAL†, M. F. ARGUS* AND J. C. ARCOS*

*From the *Department of Medicine, Tulane Medical Center and Seamen's Memorial Research Laboratory, US Public Health Service Hospital, New Orleans, Louisiana and the †Department of Biology, Southern Methodist University, Dallas, Texas*

Received 18 April 1977 Accepted 20 July 1977

**Summary.—During diethylnitrosamine (DEN) administration, a distinctive difference was observed between rats and guinea-pigs in the sequence of ultrastructural changes in the hepatic endoplasmic reticulum (ER). In DEN-induced hepatic tumour cells in the guinea-pig there was extensive proliferation of the rough ER, while the smooth ER was quite sparse; in the premalignant liver the opposite was noted. This is in contrast to the rat, in which administration of either DEN or 3′-methyl-4-dimethylaminoazobenzene (3′-Me-DAB) brings about, in both premalignant and malignant hepatic tissue, proliferation of the smooth ER and sparsity of the rough ER. Yet, as in the rat, the number of ribosomes on the outer surface of the guinea-pig liver rough ER is greatly reduced and this is paralleled by a 49% decrease of the RNA/protein ratio as early as 4 weeks of nitrosamine administration. The decrease of RNA/protein ratio and ultrastructurally observed loss of ribosomes from the ER, following nitrosamine administration, correlate with a decrease of photometric response of microsomal suspensions to the sulphydryl probe, p-chloromercuribenzoate. While azo-dye-reductase activity is higher in untreated rats than in untreated guinea-pigs, feeding 3′-Me-DAB for 6 weeks brings about a 76% decrease in the rat, but no significant decrease in the guinea-pig, which is refractory to azo-dye carcinogenesis. Thus, the ability of the liver to inactivate the dye is greatly decreased in the rat, but not in the guinea-pig, as administration progresses toward the threshold dose for tumorigenesis. On the other hand, constitutive levels of nitrosamine dealkylase are identical in the 2 species and remain essentially unchanged following administration of DEN for 10 weeks. Inasmuch as nitrosamine dealkylation represents activating metabolism, this provides a rationale for the comparable susceptibility of the rat and guinea-pig to DEN carcinogenesis. Of the 2 enzymes in the 2 species, it is only azo-dye reductase in the guinea-pig which appears to be unregulated by glucose repression, since starvation brings about no change in this activity. Starvation-induced increase of azo-dye reductase in the rat is not influenced by administration of 3′-Me-DAB and only slightly by DEN. The starvation-induced increase of nitrosamine dealkylation is abolished, however, in both species by administration of DEN but only slightly decreased by 3′-Me-DAB.**

GUINEA-PIGS have long been known to be totally resistant to the hepatocarcinogenic action of aminoazo dyes and aromatic amines. The resistance of the guinea-pig to the potent hepatic carcinogen, 3′-methyl-4-dimethylaminoazobenzene (3′-Me-DAB) remains unimpaired even when administration of the dye at 0·12% dietary level is combined with partial hepatectomy, a powerful cocarcinogenic stimulus (Gosch, Arcos and Argus, 1970). On the other hand, in the rat, admini-

Correspondence to Dr Joseph C. Arcos, Seamens' Memorial Research Laboratory, US Public Health Service Hospital, 210 State Street, New Orleans, Louisiana., 70118, USA.

stration of a 0·06% dietary level of 2-Me-DAB (an isomer of 3′-Me-DAB, inactive or marginally active in nonoperated rats) brings about an 80% tumour incidence when combined with partial hepatectomy (Warwick, 1967). In contrast to aminoazo dyes, rats and guinea-pigs show similarly high susceptibility to nitrosamine carcinogens, in particular diethylnitrosamine (DEN) [compare Argus and Hoch-Ligeti, 1961, 1963].

The refractoriness of the guinea-pig to the carcinogenic action of amine-type carcinogens, such as the azo dyes and aromatic amines, has been attributed by some investigators to the limited ability of this species to convert these agents to their N-hydroxy derivatives. Miller, Miller and Enomoto (1964) have shown that in the guinea-pig the N-hydroxy derivative of 2-acetylaminofluorene (AAF) induces adenocarcinomas of the small intestine (upon feeding) and sarcomas (upon injection) whereas AAF is inactive under these conditions. The carcinogenicity of N-hydroxy derivatives of aminoazo dyes in the guinea-pig does not appear to have been tested. However, the relative inability of the guinea-pig to N-hydroxylate, as a basis for its refractoriness to amine and azo-dye carcinogenesis, is not universally accepted (*e.g.*, Uehleke, 1969*a*,*b*). In fact, Kiese and Wiedemann (1968) attributed this refractoriness to the very rapid elimination of N-hydroxy derivatives from guinea-pig tissues. Moreover, many tissues, including the mucosa of the urinary bladder (which is not usually a target of the carcinogenic action of AAF in rats) have the ability to N-hydroxylate (Uehleke, 1969*b*). In the guinea-pig, the liver is refractory even when the N-hydroxy derivative of AAF is fed (Miller *et al.*, 1964). The view that this may be attributable to the guinea-pig's very low hepatic sulphotransferase activity (Miller and Miller, 1969) appears to be in contradiction with the finding that the mammary gland and the sebaceous gland of the external auditory canal of the rat, tissues highly susceptible to carcinogenesis by AAF and its N-hydroxy derivative, are devoid of sulphotransferase activity (Irving, Janss and Russell, 1971) regarded to be the ultimate metabolic activation step of amine-type carcinogens.

In view of these observations, it is possible that the differential susceptibility of the rat and guinea-pig to the carcinogenic action of amine-type carcinogens is determined to a large extent by metabolic pathways leading to inactive products and competing with N-hydroxylation. For aminoazo dyes, such a pathway of detoxification *par excellence* is the microsomal mixed-function oxidase, azo reductase, splitting the molecule into inactive halves. For dialkylnitrosamine carcinogens such pathway(s) leading to inactive products appear to be unknown, since the only clearly identified metabolic step of these compounds is a monodealkylation, yielding the respective aldehyde (*e.g.*, Arcos *et. al.*, 1976) whereas the remainder of the molecule gives rise non enzymatically to the ultimate carcinogen, carbonium ion (*e.g.*, Magee *et al.*, 1975).

This report describes an investigation of the comparative activities of azo-dye reductase and nitrosamine dealkylase in normal as well as azo-dye- and nitrosamine-fed rats and guinea-pigs, together with the electron microscopic alterations in the guinea-pig liver during administration of the nitrosamine. The differential susceptibility of the guinea-pig to azo dye and nitrosamine carcinogenesis is correlated with a spectrophotometric study of microsomal-membrane alterations during administration of the two agents, using an -SH reactor membrane probe.

### MATERIALS AND METHODS

*Care of animals and administration of carcinogens.*—English short-haired albino male guinea-pigs, initial weight 150–230 g (Perfection Breeders, Douglasville, PA, USA) and Sprague-Dawley male rats, initial weight 50–60 g (Holtzman Co., Madison, WI, USA) were used; the guinea-pigs were housed 1, and the rats 2, to a cage. The guinea-pigs were maintained on Purina rabbit chow pellets or ground rabbit chow into which 3′-Me-DAB was incorporated at the level of 0·12%; these animals received 80–100 mg ascorbic acid daily, freshly dissolved in the drinking water. The rats received laboratory chow, or an 18% casein-containing semi-synthetic diet (Arcos, Argus and Wolfe, 1968) or these diets into which 3′-Me-DAB was incorporated at the level of 0·06%.

Regarding the choice of the nitrosamine used in this study, both dimethylnitrosamine (DMN) and diethylnitrosamine (DEN) have been shown to be hepatic carcinogens in the guinea-pig (LePage and Christie, 1969; Argus and Hoch-Ligeti, 1963). However, the choice of the nitrosamine was restricted by the fact that despite the considerable similarities between the dealkylation reactions of dialkylnitrosamines (Phillips *et al.*, 1975; Weekes and Brusick, 1975; Bartsch, Malaveille and Montesano, 1975; Arcos *et al.*, 1976) the 48h acute toxicity of DMN was found to be over 5 × that of DEN in the guinea-pig (DMN $LD_{50}$: 126 ± 39 mg/kg and DEN $LD_{50}$: 692 ± 46 mg/kg, with fiducial limits set at 95% probability). On the other hand, the acute toxicity of DEN is similar in the rat and the guinea-pig and could, therefore, be administered to the two species at similar maximal levels (per kg body weight) tolerated chronically. Thus, in the present study DEN was administered to both guinea-pigs and rats in the drinking water: 0·023 ml DEN/l for rats and 0·042 ml DEN/l for guinea-pigs. The average daily intake of DEN was 0·65 mg/rat and 1·2 mg/guinea-pig. Guinea-pigs receiving DEN were fed rabbit chow and were given the ascorbic acid supplement by stomach tube, at the level of 100 mg 3 × weekly. Rats receiving DEN were maintained on laboratory chow.

The two carcinogens were administered up to the respective lengths of time necessary to reach a cumulative dose for a defined onset of tumorigenesis (in terms of a 10 to 50% tumour incidence) in the susceptible species. Thus, 3′-Me-DAB was fed for 6 weeks to both species, a period of administration selected on the basis of a 3′-Me-DAB tumourigenesis dose-response curve established in the rat (Arcos, Griffith and Cunningham, 1960), the susceptible species. Moreover, in the guinea-pig, refractory to azo-dye carcinogenesis, the dietary level of the dye was doubled in order to maximize any possible effect on the biochemical parameters studied. DEN was administered to both species for 10 weeks, a length of time selected on the basis of a DEN tumorigenesis dose-response curve in the guinea-pig (Arcos, Argus and Mathison, 1969) the only nitrosamine for which a dose-response curve has been determined in this species. Moreover, the DEN dose response of rat and guinea-pig appears to be similar. In rats (average initial weight 92 g) receiving an average dose of 0·55 mg 5 × weekly, the first tumour appeared at 22 weeks (Argus and Hoch-Ligeti, 1961) whereas in guinea-pigs (average initial weight 255 g) receiving an average daily dose of 1·78 mg, the first tumour appeared at 16 weeks (Argus and Hoch-Ligeti, 1963).

*Electron microscopy.*—Small pieces (∼1 $mm^3$) of tissue were fixed in phosphate-buffered 4% glutaraldehyde at 4°C for 1½ h, washed in phosphate buffer and refixed in in phosphate-buffered 1% osmium tetroxide for 1 h. Tissues were dehydrated in an ascending series of ethanol concentrations and embedded in Maraglas. Thin sections were cut with an LKB Ultrotome microtome and stained with uranyl acetate and lead citrate. A Hitachi HU11B electron microscope was used for observations.

*Mixed-function oxidase determinations.*—An average 1g sample of liver was used for the azo-dye-reductase assay; from the remainder of the liver, the microsomes were isolated for the DMN-demethylase assay. Azo-reductase activity was determined (in triplicate) using a 10% homogenate in 0·88M sucrose, following Chauveau and Decloitre (1968) with 3′-Me-DAB or 2-Me-DAB as substrate, and using the corresponding reference curve. Incubation was for 30 min. Activity is expressed as nmol azo dye reduced/g liver/min.

Using DMN-demethylase activity as a measure of nitrosamine dealkylation, 4–7 individual values were averaged. The isolation of microsomes, dealkylation reaction and the determination of HCHO by the Nash reaction were as previously described (Venkatesan, Arcos and Argus, 1968, 1970*b*) except that in the present study the final volumes of the dealkylase medium and of all components were halved. Microsomal protein was determined following Lowry *et al.* (1951) and RNA following Schneider (1957) with yeast RNA (purified; Sigma) as standard.

*Determination of microsomal absorbance changes.*—Microsomes were isolated as follows. The livers were homogenized (40% w/v homogenate) at 2–4 °C in glass-teflon homogenizer in 0·25M sucrose + 0·001M ethylenediaminetetraacetate. Nuclei and mitochondria were sedimented at 15,000 *g* for 15 min. The supernatant fraction was recentrifuged at 20,000 *g* for 12 min to remove possible mitochondrial contamination. The final supernant was centrifuged at 105,000 *g* for 50 min. The resulting pellet was washed by resus-

FIG. 1.—Hepatic cell from a normal control guinea-pig showing part of a nucleus (N). The cisternae of rough endoplasmic reticulum (RER) are in stacked form or in isolated fragments. Smooth endoplasmic reticulum (SER), glycogen (Gl), Golgi complex(G), lysosome (L), and mitochondria (M) appear essentially as identified. × 26000.

FIG. 2.—Hepatocyte from premalignant guinea-pig liver (10½ weeks of DEN) showing a proliferation of smooth endoplasmic reticulum (SER). Cisternae of rough endoplasmic reticulum (arrow) occur singly, usually surrounding the mitochondria. × 21000.

pension and homogenization in 0·25M sucrose containing no ethylenediaminetetraacetate, and recentrifuged for 60 min. The final pellet was resuspended in 0·25M sucrose so that 1 ml contained the microsomes from 2 g of tissue. This standard stock suspension was kept in ice and used within 20 min.

The assay system, adapted from Robinson (1967) consisted of appropriate aliquots of microsomal stock suspension added to 50 mM Tris-HCl (pH 7·3) plus 0·05 ml 3mM ATP or ADP or 0·05 ml water (final volume 1·05 ml), so that the initial absorbance was between 0·290 and 0·310. Absorbance decrease was recorded against a Tris-HCl blank at 10, 20, 30 and 45 min at 25°C. At 45 min 0·05 ml (0·1mM) p-chloromercuribenzoate (p-CMB) was added to each cuvette and the absorbance read immediately and at 50, 60, 65 and 70 min. To insure homogeneity of the suspensions, the cuvettes were always tilted before reading. A set of duplicate cuvettes was carried through the assay, adding 0·05 ml water at 45 min so that a dilution factor for the addition of p-CMB could be determined. The percentage microsomal absorbance change in the two phases of the assay was calculated by relating the absorbances at the time intervals to the respective initial values. When the effect of carcinogen administration on p-CMB-induced absorbance decrease was studied, 6 control determinations and 3 determinations with DEN- or 3′-Me-DAB-administered guinea-pigs were carried out.

### RESULTS

### *DEN-induced ultrastructural alterations in guinea-pig liver*

The fine structure of the control guinea-pig hepatocytes (Fig. 1) is essentially similar to that of the rat hepatocytes previously described by Bruni and Porter (1965). Rough endoplasmic reticulum (RER) is well developed, occurring as stacks of parallel cisternae as well as isolated units. Tubules of smooth endo-

FIG. 3.—Hepatic cell from tumour-bearing guinea-pig liver showing the formation of an extensive network of RER cisternae (arrow). The number of ribosomes attached to the RER membrane is reduced. Free ribosomes in polysomal (R) configurations are numerous. × 21000.

plasmic reticulum (SER) are seen primarily in the glycogen-rich areas of the cytoplasm. Glycogen is predominantly of α type. Mitochondria are randomly distributed. Lysosomes and Golgi components most frequently occur near the bile canaliculi.

*Premalignant liver.*—The hepatocytes of guinea-pigs fed DEN for $7\frac{1}{2}$ and $10\frac{1}{2}$ weeks and from the non-tumour region of the liver at $20\frac{1}{2}$ weeks, appear to have undergone general dedifferentiation (Fig. 2). The elaborate arrays of parallel RER cisternae are absent. The cisternae are isolated and frequently lie in close apposition to the mitochondria. Glycogen appears to be reduced in many cells, with an accompanying prominence and proliferation of SER. Free ribosomes occur with greater frequency. Nuclei are generally large and rounded.

*Tumour cells.*—These are mostly polygonal in shape, with prominent round nuclei, and show strong resemblance to hepatocytes. Bile canaliculi are frequently observed. One of the most striking features of the tumour cells is the great proliferation of RER which forms an extensive but irregularly disposed interconnective network of cisternae (Fig. 3).

FIG. 4.—A guinea-pig hepatic-tumour cell showing annulated lamellae (AL) and the continuation of the lamellae with RER (arrow). RER forms a network. × 26500.

FIG. 5.—A guinea-pig hepatic-tumour cell showing disruption of the nuclear membrane (arrow). × 14000.

The parallel arrays of RER cisternae were never encountered. RER membranes are extensively distributed within the cytoplasm and show frequent continuities with the nuclear envelope. The number of ribosomes on the outer surface of RER membranes is greatly reduced. The free ribosomes are abundant and are mainly arranged in polysomal configurations (Fig. 3).

SER is quite sparse. Annulate lamellae consisting of several parallel double membranes, which are frequently interrupted by pores or annuli, are occasionally seen (Fig. 4). They are continuous with RER at their periphery.

Mitochondria exhibit several variations in structure (*e.g.*, reduced matricial density and reduction, absence or dilation of the cristae; or the cristae may be arranged in stacks). Glycogen is absent in many tumour cells; in others it is sparse and randomly distributed. The Golgi complex is often well developed. The number of Golgi complexes in tumour cells is considerably increased and up to 5 Golgi complexes could be seen in a single section of a cell. The Golgi complexes are randomly distributed, compared to their pericanicular localization in the normal cell.

Interruptions in the nuclear envelope were observed (Fig. 5). Lipid droplets and lysosomes were abundant in some cells.

*Change in mixed-function oxidase levels*

During the period of administration of 3′-Me-DAB and DEN to rats and guinea-pigs, the amount of microsomal protein/g tissue underwent considerable variations. Following a 6-week administration of 3′-Me-DAB the amount of microsomal protein/g tissue was decreased by 17% ($P < 0{\cdot}01$) in the rat, but increased by 34·6% ($P < 0{\cdot}01$) in the guinea-pig. However, following a 10-week administration of DEN this parameter was changed in the opposite way; there was a 15% increase of microsomal protein in the rat ($0{\cdot}01 > P > 0{\cdot}001$) and a 12·4% decrease in the guinea-pig ($0{\cdot}05 > P$

TABLE I.—*Effect of Carcinogen Administration on Azo-dye-reductase and Nitrosamine-dealkylase Activities*

| Animal | Control | | 3′-Me-DAB for 6 weeks | | DEN for 10 weeks | |
|---|---|---|---|---|---|---|
| | Azo-reductase activity ‡ | Nitrosamine-dealkylase activity § | Azo-reductase activity ‡ | Nitrosamine-dealkylase activity § | Azo-reductase activity ‡ | Nitrosamine-dealkylase activity § |
| Rat * | 37·5 ± 4·5 ‖<br>43·0 ± 3·9 ¶ | 7·9 ± 0·5 | 10·2 ± 0·6<br>($P < 0·001$) | 5·5 ± 0·4<br>($0·01 > P > 0·001$) | 52·8 ± 3·4<br>($0·05 > P > 0·02$) | 8·5 ± 0·6<br>($0·50 > P > 0·40$) |
| Guinea-pig † | 25·0 ± 4·6 | 7·7 ± 0·7 | 16·9 ± 1·4<br>($0·20 > P > 0·10$) | 11·8 ± 1·0<br>($0·01 > P > 0·001$) | 31·2 ± 3·5<br>($P \sim 0·30$) | 6·0 ± 1·0<br>($0·20 > P > 0·10$) |

* 10 weeks old at killing if on 3′-Me-DAB and 13 weeks old if on DEN

† 33 weeks old at killing on both 3′-Me-DAB and DEN

‡ nmol 3′-Me-DAB reduced/g tissue/min; each value is the average of 3 determinations ± s.e.

§ nmol HCHO formed in DMN demethylation/g tissue/min; each value is the average of 4–7 determinations ± s.e.

‖ Animals fed laboratory chow

¶ Animals fed the semisynthetic diet and serving as the reductase controls for rats fed 3′-Me-DAB. The difference between the two control values was not significant ($P \sim 0·40$).

$>$ 0·02). Administration of DEN to the guinea-pig brought about a decrease of total microsomal RNA, which is consistent with the great reduction in the number of ribosomes on the outer surface of the RER membrane (in "DEN-induced ultrastructural alterations . . . "). Already after 4 weeks the RNA/protein ratio decreased by 49% ($P < 0·001$). Administration of 3′-Me-DAB for as long as 15 weeks did not affect the RNA/protein ratio.

Table I shows that the level of azo-dye-reductase activity is higher in control rats than in control guinea-pigs, which is unexpected, since the guinea-pig is totally resistant to azo-dye carcinogenesis (Gosch *et al.*, 1970). However, feeding 3′-Me-DAB to both species produced a 76% decrease in reductase activity in the rat, but only a nonsignificant 32% decrease in the guinea-pig. DEN administration left reductase activity essentially unchanged in the guinea-pig, but increased activity by 40·8% in the rat. Table I also shows that the level of nitrosamine-dealkylase activity was essentially identical in both species, and was unchanged by administration of DEN. Following feeding of 3′-Me-DAB, however, dealkylase activity was decreased by 30·4% in the rat and increased by 53·2% in the guinea-pig; this may be attributed primarily to the effect of the dye on the amount of microsomes in the tissue (see above). Only the azo-dye-reductase values obtained with 3′-Me-DAB as substrate are presented in Table I, since enzyme activities were essentially the same when 2-Me-DAB was used.

Figs. 6 and 7 present the effect of 24 h starvation on azo-dye-reductase and nitrosamine-dealkylase activities. Starvation, by way of the release of carbohydrate repression of enzyme synthesis (Peraino and Pitot, 1964; Tschudy *et al.*, 1964; Young, Shrago and Lardy, 1964) is known to bring about in the rat a substantial increase in the level of azo-dye reduction (Jervell, Christoffersen and Morland, 1965) and DMN dealkylation (Venkatesan, Arcos and Argus, 1970*a*). Fig. 6 shows that in the rat chronic administration of neither 3′-Me-DAB nor

FIG. 6.—Effect of carcinogen administration and starvation on hepatic azo-dye-reductase activity in the rat and guinea-pig. 3′-Methyl-4-dimethylaminoazobenzene (3′-Me-DAB) was administered for 6 weeks and diethylnitrosamine (DEN) for 10 weeks. Starvation, where indicated, was for 24 h before sacrifice. 3′-Me-DAB was used as reductase substrate. Fed: □; starved: ▩. The bars represent the means of 3 individual determinations ± s.e.

FIG. 7.—Effect of carcinogen administration and starvation on hepatic nitrosamine-dealkylase activity in the rat and guinea-pig. 3′-Me-DAB was administered for 6 weeks and DEN for 10 weeks. Starvation, where indicated, was for 24 h before sacrifice. Dimethylnitrosamine was used as dealkylase substrate. Fed: □; starved: ▩. The bars represent the means of 4–7 individual determinations ± s.e.

DEN appreciably influences the extent of starvation-induced increase of the reductase. The significance of the difference between the starved and non-starved values is $0{\cdot}01 > P > 0{\cdot}001$ or better in the 4 instances. On the other hand, in the guinea-pig, the starvation-induced increase of the reductase is totally absent in the control as well as in 3′-Me-DAB- or DEN-administered animals.

Fig. 7 shows that the starvation-induced increase of nitrosamine dealkylase in the rat is not affected by chronic administration of 3′-Me-DAB, but is essentially suppressed by DEN administration. The significance of the difference between the starved and non-starved values in both control and 3′-Me-DAB-fed rats is $0{\cdot}01 > P > 0{\cdot}001$ or better, and for the DEN-fed rats is $0{\cdot}20 > P > 0{\cdot}10$. However, in the guinea-pig (unlike the absence of starvation effect on azo-dye reductase seen in Fig. 6) Fig. 7 shows that nitrosamine dealkylase is induced 65% by starvation, and that this increase is reduced to 31% by 3′-Me-DAB administration. The significance of the difference between starved and non-starved values in control and 3′-Me-DAB-fed animals is $0{\cdot}05 > P > 0{\cdot}02$ and $0{\cdot}02 > P > 0{\cdot}01$, respectively. Administration of DEN abolished the starvation-induced dealkylase increase also in the guinea-pig, since the slight increase is not significant ($P = 0{\cdot}10$).

*ER membrane alteration*

The combined effect of nucleotides and of the sulphydryl reactor, p-CMB, on absorbance change in guinea-pig liver microsomes is shown in Fig. 8. The presence of ATP in the medium did not alter significantly the absorbance change at 45 min; the presence of ADP caused, however, a 35% decrease ($P < 0{\cdot}001$) below the control. Addition of p-CMB at the end of the 45-min period brought about an immediate drop of absorbance; the presence of ATP or ADP reduced this drop by 50% or more ($P < 0{\cdot}001$).

Administration of DEN for 8 weeks substantially reduced the p-CMB-produced

FIG. 8.—Representative experiment showing the effect of p-chloromercuribenzoate (p-CMB) on the rate of absorbance decrease of guinea-pig liver-microsome suspensions in a hypotonic medium, in the presence of ADP or ATP. The assay system consisted of 1·05 ml 50mM Tris-HCl (pH 7·3); the nucleotides when present were at the level of 150 nmol. p-CMB was added at the time indicated as a 0·05 ml aliquot of a 0·1mM solution. Parallel control cuvettes, to which 0·05 ml water was added at 45 min, were carried through the assay for determining the dilution factor due to p-CMB addition.

decrease of absorbance (Table II). Although the presence of the nucleotides decreased the extent of the absorbance drop, the effect of DEN administration remained significant. Interestingly, feeding DEN for 1 to 15 weeks did not affect the microsomal absorbance change recorded *prior* to p-CMB addition. The microsomal absorbance change observed under these different conditions remained unaltered after feeding 3′-Me-DAB for 15 weeks.

### DISCUSSION

### *Ultrastructural modifications*

In DEN-induced hepatic tumour cells in the guinea-pig the RER was found to have undergone extensive proliferation, forming a network of cisternae, rather than occurring in stacked form or in isolated and fragmented form; the SER is, however, quite sparse in these tumour cells. This is in contrast with DEN-induced liver tumours in the rat, in which increase of the SER and fragmentation and disaggregation of the RER were observed (Svoboda and Higginson, 1968).

It is striking that in the guinea-pig the decrease of SER and proliferation of RER in the tumour cells is preceded by an increase of the SER and decrease and fragmentation of the RER in the hepatocytes of premalignant liver. If the distribution of mixed-function oxidases in hepatic ER is similar in the rat and

TABLE II.—*Effect of Diethylnitrosamine Administration on p-Chloromercuribenzoate-induced Absorbance Decrease of Suspensions of Guinea-pig Liver Microsomes*

| Nucleotide in the assay system * | % decrease † Control | % decrease † 8 weeks DEN-administration | Significance of difference |
|---|---|---|---|
| None | 5·7 ± 0·3 | 4·1 ± 0·2 | P ~ 0·05 |
| ADP | 3·1 ± 0·3 | 1·4 ± 0·2 | *P* < 0·001 |
| ATP | 1·5 ± 0·2 | 0·6 ± 0·4 | 0·10 > *P* > 0·05 |

* 1·05 ml 50mM Tris-HCl (pH 7·3) containing no nucleotide or 150 nmol ADP or ATP. At 45 min (see Fig. 8) 0·05 ml 0·1mM p-CMB was added, and the absorbance change recorded immediately and at intervals up to 70 min; the same volume of water was added to control cuvettes carried through the assay for determining the dilution factor due to p-CMB addition.

† Of absorbance recorded immediately after p-CMB addition. Each control and each experimental value represents the mean ± s.e. of 6 and 3 experiments, respectively.

guinea-pig, then the reduction of SER in guinea-pig hepatic tumour cells may reflect a decreased capacity to metabolize DEN. It has been documented that hepatic tumours have decreased capacity to metabolize nitrosamines (Brouwers and Emmelot, 1960).

The opposite responses of rats and guinea-pigs to administation of 3′-Me-DAB or DEN in terms of microsomal protein/g tissue (*vide* "Change in mixed-function oxidase levels" above) may be due to a differential effect of the two carcinogens on the relative proliferative rates of the SER and RER.

The mitochondrial changes noted, reflecting disturbances of mitochondrial function, have also been observed in hepatocellular carcinomas by others (Svoboda, 1964; Ma and Webber, 1966). Arcos *et al.* (1969) reported that administration of the minimum effective tumour dose of DEN brings about dramatic changes in the swelling response of guinea-pig liver mitochondria; no changes are seen following administration of 3′-Me-DAB (Arcos *et al.*, 1961).

*Mixed-function-oxidase activities*

Administration of carcinogens, including aminoazo dyes and nitrosamines, is well known to lower, by varying degrees, different hepatic mixed-function-oxidase activities in the rat (*e.g.*, Baldwin and Barker, 1965; Smuckler, *et al.*, 1967; Ketterer, *et al*, 1968; Stevenson and Greenwood, 1968; Friedman, *et al.*, 1976). Azo-dye reductase, which brings about the reductive cleavage of aminoazo dyes into inactive halves, may be regarded as a detoxifying enzyme *par excellence;* therefore, the reductase level maintained in the tissue during dye administration appears to represent a rate-limiting factor of carcinogenesis. The present results show that while azo-dye-reductase activity is higher in untreated rats than in untreated guina-pigs, feeding 3′-Me-DAB until the critical dose is reached brings about considerable decrease of enzyme activity in the rat, but no significant decrease in the guinea-pig. This indicates that the ability of the liver to inactivate the dye gradually decreases in the rat but not in the guinea-pig as intake progresses toward the threshold dose for tumorigenesis.

Nitrosamine dealkylation is regarded as the single activating step of dialkylnitrosamine carcinogens (*e.g.*, Magee *et al.*, 1975). Hence, it appears significant that the nitrosamine-dealkylase levels remained unimpaired in both species following administration of DEN up to the threshold dose for carcinogenesis. It is noteworthy that the dealkylase levels were identical in untreated animals of the two species, consistent with the virtually identical susceptibility of the rat and guinea-pig to DEN carcinogenesis (compare Argus and Hoch-Ligeti, 1961, 1963). Detoxifying pathway(s) of nitrosamine metabolism comparable in role to azo-dye reductase are not known.

Interestingly, of the two mixed-function oxidases in the two species only azo-dye reductase in the guinea-pig is unresponsive to glucose repression, since starvation brings about no change in its activity (Fig. 6). Azo-dye reductase in the rat (Fig. 6) as well as nitrosamine dealkylase in both the rat and guinea-pig (Fig. 7) are, on the other hand, substantially induced by starvation. It is possible that the lack of glucose repression of the reductase in the guinea-pig may be related to the sustained level of this enzyme during 3′-Me-DAB administration and, hence, to the resistance of this species to azo-dye carcinogenesis.

*Sulphydryl- reactor- sensitive membrane alteration*

The present results are in general agreement with the conclusion of Robinson (1967) in that ADP and ATP markedly influence the reactivity of membrane sulphydryl groups toward p-CMB and thereby the photometrically detected structural change. However, at variance with his findings, we observed that ADP, and to a lesser extent ATP, inhibited rather than potentiated the effect of p-CMB (Fig. 8). Administration of DEN brought about a large percentage decrease of the response to p-CMB, in the absence or presence of the nucleotides (Table II).

There is evidence that the polyribosome particles are bound to the ER *via* sulphydryl-disulphide interchange (Williams and Rabin, 1969, 1971; Williams and Parry, 1975) and that the degranulation of the ER in acute *in vitro* systems by reactive metabolic intermediates of carcinogens brings about an "unmasking" of sulphydryl-disulphide interchange activity (Williams and Rabin, 1969, 1971; Williams, Clark and Rabin, 1973; Williams and Parry, 1975). The presently observed decrease of reactivity toward the -SH reactor, p-CMB, following chronic DEN administration, suggests two alternatives: (a) that during *in vivo* degranulation the -SH groups which arise undergo intramembrane recombination, or combination with reactive metabolic intermediates of the carcinogens (*cf.* Craddock, 1964) or (b) that, with the progression of the neoplastic change, the mechanism governing the osmotic behaviour of the ER membrane becomes increasingly segregated from the sites of sulphydryl-disulphide interchange activity.

This investigation was supported by grants CA-13206 and CA-15111 from the National Cancer Institute and Grant No. 922M from the Council for Tobacco Research, U.S.A. Joseph C. Arcos was the recipient of a Faculty Research Award from the American Cancer Society. We are indebted to Dr Robert D. Yates and Dr David W. Fredericksen for helpful criticism.

### REFERENCES

ARCOS, J. C., ARGUS, M. F. & MATHISON, J. B. (1969) Hepatic Carcinogenesis Threshold and Biphasic Mitochondrial Swelling Response in the Guinea-Pig During Diethylnitrosamine Administration. *Experientia*, **25**, 296.

ARCOS, J. C., ARGUS, M. F. & WOLF, G. (1968) In *Chemical Induction of Cancer*, Vol. I, New York: Acadamic Press. pp. 375 and 450.

ARCOS, J. C., BRYANT, G. M., PASTOR, K. M. & ARGUS, M. F. (1976) Structural Limits of Specificity of Methylcholanthrene-repressible Nitrosamine N-Dealkylases. Inhibition by Analog Substrates. *Z. Krebsforsch.*, **86**, 171.

ARCOS, J. C., GOSCH. H. H. & ZICKAFOOSE, D. (1961) Fine Structural Alterations in Cell Particles During Chemical Carcinogenesis. III. Selective Action of Hepatic Carcinogens Other than 3′-Methyl-4-dimethylaminoazobenzene on Different Types of Mitochondrial Swelling. Effect of Stimulated Liver Growth. *J. biophys. biochem. Cytol.*, **10**, 23.

ARCOS, J. C., GRIFFITH, G. W. & CUNNINGHAM, R. W. (1960) Fine Structural Alterations in Cell Particles During Chemical Carcinogenesis. II. Further Evidence for their Involvement in the Mechanism of Carcinogenesis. The Swelling of Rat Liver Mitochondria During Feeding of Amino Azo Dyes. *J. biophys. biochem. Cytol.*, **7**, 49.

ARGUS, M. F. & HOCH-LIGETI, C. (1961) Comparative Study of the Carcinogenic Activity of Nitrosamines. *J. natn. Cancer Inst.*, **27**, 695.

ARGUS, M. F. & HOCH-LIGETI, C. (1963) Induction of Malignant Tumours in the Guinea-pig by Oral Administration of Diethylnitrosamine. *J. natn. Cancer Inst.*, **30**, 533.

BALDWIN, R. W. & BARKER, C. R. (1965) Influence of Aminoazo Dyes on Drug Metabolism in Rat Liver. *Br. J. Cancer*, **19**, 565.

BARTSCH, H., MALAVEILLE, C. & MONTESANO, R. (1975) *In vitro* Metabolism and Microsome-mediated Mutagenicity of Dialkylnitrosamines in Rat, Hamster and Mouse Tissues. *Cancer Res.*, **35**, 644.

BROUWERS, J. A. J. & EMMELOT, P. (1960) Microsomal N-Demethylation and the Effect of the Hepatic Carcinogen Dimethylnitrosamine on Amino Acid Incorporation into the Proteins of Rat Livers and Hepatomas. *Expl Cell Res.*, **19**, 467.

BRUNI, C. & PORTER, K. R. (1965) The Fine Structure of Parenchymal Cells of the Normal Rat Liver. I. General Observations. *Am. J. Path.*, **46**, 691

CHAUVEAU, J. & DECLOITRE, F. (1968) Mise en Evidence chez le Rat d'un Facteur Génétique dans la Cancérisation par le Diméthylaminoazobenzène. *Int. J. Cancer*, **3**, 88.

CRADDOCK, V. M. (1964) Reaction of the Carcinogen Dimethylnitrosamine with Liver Thiol Groups *In vivo. Biochem. J.*, **90**, 33P.

FRIEDMAN, M. A., SANDERS, V. & WOODS, S. (1976) Suppression of Dimethylnitrosamine Metabolism and Toxicity by Nitrososarcosine and Other Nitrosamines. *Toxicol. appl. Pharmacol.*, **36**, 395.

GOSCH, H. H., ARCOS, J. C. & ARGUS, M. F. (1970) On the Unimpairable Resistance of the Guinea-pig to Dietary Amino Azo Dye Hepatocarcinogenesis. *Z. Krebsforsch.*, **73**, 215.

IRVING, C. C., JANSS, D. H. & RUSSELL, L. T. (1971) Lack of N-Hydroxy-2-acetylaminofluorene Sulfotransferase Activity in the Mammary Gland and Zymbal's Gland of the Rat. *Cancer Res.*, **31**, 387.

JERVELL, K. F., CHRISTOFFERSEN, T. & MORLAND J. (1965) Studies on the 3-Methylcholanthrene Induction and Carbohydrate Repression of Rat Liver Dimethylaminoazobenzene Reductase. *Arch. Biochem. Biophys.*, **111**, 15.

KETTERER, B., ROSS-MANSELL, P. & DAVIDSON, H. (1968) The Effect of 4-Dimethylaminoazobenzene and Corn Oil on Azo-Dye Reductase in the Rat Liver. *Biochem. J.*, **107**, 15P

KIESE, M. & WIEDEMANN, I. (1968) Elimination of N-Hydroxy Arylamines from the Blood of Guinea-pigs. *Biochem. Pharmacol.*, **17**, 1151.

LEPAGE, R. N. & CHRISTIE, G. S. (1969) Induction of Liver Tumours in the Guinea-pig by Feeding Dimethylnitrosamine. *Pathology*, **1**, 49.

LOWRY, O. H., ROSEBROUGH, N. J., FARR, A. L. & RANDALL, R. (1951) Protein Measurement with the Folin Phenol Reagent. *J. biol. Chem.*, **193**, 265.

MA, M. H. & WEBBER, A. J. (1966) Fine Structure of Liver Tumours Induced in the Rat by 3′-Methyl-4-dimethylaminoazobenzene. *Cancer Res.*, **26**, 935.

MAGEE, P. N., NICOLL, J. W., PEGG, A. E. & SWANN, P. F. (1975) Alkylating Intermediates in Nitrosamine Metabolism. *Biochem. Soc. Trans.*, **3**, 62.

MILLER, E. C., MILLER, J. A. & ENOMOTO, M. (1964) The Comparative Carcinogenicities of 2-Acetylaminofluorene and Its N-Hydroxy Metabolite in Mice, Hamsters, and Guinea-Pigs. *Cancer Res.*, **24**, 2018.

MILLER, J. A. & MILLER, E. C. (1969) Metabolic Activation of Carcinogenic Aromatic Amines and Amides via N-Hydroxylation and N-Hydroxy-esterification and its Relationship to Ultimate Carcinogens as Electrophilic Reactants. In *Jerusalem Symp. Quant. Chem. & Biochem. No.* 1 *Physico-Chemical Mechanisms of Carcinogenesis.* Eds. E. D. Bergmann & B. Pullman. Jerusalem: Israel Acad. Sci. & Humanities.

PERAINO, C. & PITOT, H. C. (1964) Studies on the Induction and Repression of Enzymes in Rat Liver. II. Carbohydrate Repression of Dietary and Hormonal Induction of Threonine Dehydrase and Ornithine δ-Transaminase. *J. biol. Chem.*, **239**, 4308.

PHILLIPS, J. C., LAKE, B. G., MINSKI, M. J., GANGOLLI, S. D. & LLOYD, A. G. (1975) Studies on the Metabolism of Diethylnitrosamine in the Rat. *Biochem. Soc., Trans.* **3**, 285.

ROBINSON, J. D. (1967) Structural Changes in Microsomal Suspensions. V. Interactions with Nucleotides. *Arch. Biochem. Biophys.*, **118**, 649.

SCHNEIDER, W. C. (1957) Determination of Nucleic Acids in Tissues by Pentose Analysis. *Meth. Enzymol.*, **3**, 680.

SMUCKLER, E. A., ARRHENIUS, E. & HULTIN, T. (1967) Alterations in Microsomal Electron Transport, Oxidative N-Demethylation and Azo Dye Cleavage in Carbon Tetrachloride and Dimethylnitrosamine-induced Liver Injury. *Biochem. J.*, **103**, 55.

STEVENSON, I. H. & GREENWOOD, D. T. (1968) Inhibition of Hexobarbital Metabolism by Diethylnitrosamine. *Biochem. Pharmacol.*, **17**, 842.

SVOBODA, D. J. (1964) Fine Structure of Hepatomas Induced in Rats with p-Dimethylaminoazobenzene. *J. natn. Cancer Inst.*, **33**, 315.

SVOBODA, D. J. & HIGGINSON, J. A. (1968) Comparison of Ultrastructural Changes in Rat Liver Due to Chemical Carcinogens. *Cancer Res.*, **28**, 1703.

TSCHUDY, D. P., WELLAND, F. H., COLLINS, A. & HUNTER, G. (1964) The Effect of Carbohydrate Feeding on the Induction of δ-Aminolevulinic Acid Synthetase. *Metabolism*, **13**, 396.

UEHLEKE, H. (1969a) Toxikologische Aspekte der N-Hydroxylierung aromatischer Amine. *Arch. Pharmakol. expt. Pathol.* **263**, 106.

UEHLEKE, H. (1969*b*) General Biological Aspects of N-Hydroxylation. *Fed. Eur. Biochem. Soc. Symp.*, **16**, 97.

VENKATESAN, N., ARCOS, J. C. & ARGUS, M. F. (1968) Differential Effect of Polycyclic Hydrocarbons on the Demethylation of the Carcinogen Dimethylnitrosamine by Rat Tissues. *Life Sci.*, **7**(1), 1111.

VENKATESAN, N., ARCOS, J. C. & ARGUS, M. F. (1970*a*) Amino Acid Induction and Carbohydrate Repression of Dimethylnitrosamine Demethylase in Rat Liver. *Cancer Res.*, **30**, 2563.

VENKATESAN, N., ARGUS, M. F. & ARCOS, J. C. (1970*b*) Mechanism of 3-Methylcholanthrene-induced Inhibition of Dimethylnitrosamine-demethylase in Rat Liver. *Cancer Res.*, **30**, 2556.

WARWICK, G. P. (1967) The Covalent Binding of Metabolites of Tritiated 2-Methyl-4-dimethylaminoazobenzene to Rat Liver Nucleic Acids and Proteins, and the Carcinogenicity of the Unlabelled Compound in Partially Hepatectomized Rats. *Eur. J. Cancer*, **3**, 227.

WEEKES, U. & BRUSICK, D. (1975) *In vitro* Metabolic Activation of Chemical Mutagens. II. The Relationships Among Mutagen Formation, Metabolism and Carcinogenicity for Dimethylnitrosamine and Diethylnitrosamine in the Livers, Kidneys, and Lungs of BALB/cJ, C57BL/6J and RF/J Mice. *Mutat. Res.*, **31**, 175.

WILLIAMS, D. J., CLARK, R. P. & RABIN, B. R. (1973) The Effects of Aflatoxin $B_1$ *In vivo* on Membrane-ribosome Association. *Br. J. Cancer*, **27**, 283.

WILLIAMS, D. J. & PARRY, G. (1975) Endoplasmic Membrane as a Source and as a Target for Chemically Reactive Metabolic Intermediates. *Biochem. Soc. Trans.*, **3**, 69.

WILLIAMS, D. J. & RABIN, B. R. (1969) The Effects of Aflatoxin $B_1$ and Steroid Hormones on Polysome Binding to Microsomal Membranes as Measured by the Activity of an Enzyme Catalyzing Disulfide Interchange. *FEBS Lett.*, **4**, 103.

WILLIAMS, D. J. & RABIN, B. R. (1971) Disruption by Carcinogens of the Hormone Dependent Association of Membranes with Polysomes. *Nature, Lond.*, **232**, 102.

YOUNG, J. W., SHRAGO, E. & LARDY, H. A. (1964) Metabolic Control of Enzymes Involved in Lipogenesis and Gluconeogenesis. *Biochemistry*, **3**, 1687.